# Optimization of an Efficient Protein Extraction Protocol Compatible with Two-Dimensional Electrophoresis and Mass Spectrometry from Recalcitrant Phenolic Rich Roots of Chickpea (*Cicer arietinum* L.)

**DOI:** 10.1155/2012/536963

**Published:** 2012-10-31

**Authors:** Moniya Chatterjee, Sumanti Gupta, Anirban Bhar, Sampa Das

**Affiliations:** Division of Plant Biology, Bose Institute, Centenary Campus, P 1/12, CIT Scheme VII-M, Kankurgachi, West Bengal, Kolkata 700054, India

## Abstract

Two-dimensional electrophoresis and mass spectrometry are undoubtedly two essential tools popularly used in proteomic analyses. Utilization of these techniques however largely depends on efficient and optimized sample preparation, regarded as one of the most crucial steps for recovering maximum amount of reliable information. The present study highlights the optimization of an effective and efficient protocol, capable of extraction of root proteins from recalcitrant phenolic rich tissues of chickpea. The widely applicable TCA-acetone and phenol-based methods have been comparatively evaluated, amongst which the latter appeared to be better suited for the sample. The phenol extraction-based method further complemented with sodium dodecyl sulphate (SDS) and pulsatory treatments proved to be the most suitable method represented by greatest spot number, good resolution, and spot intensities. All the randomly selected spots showed successful identification when subjected to further downstream MALDI-TOF and MS/MS analyses. Hence, the information obtained collectively proposes the present protein extraction protocol to be an effective one that could be applicable for recalcitrant leguminous root samples.

## 1. Introduction

Presence of intricate photosynthetic machinery, cell wall and other organelles, complex primary and secondary metabolic processes, and their cellular regulation adds to the complexity of functional biology of plants. In recent years, proteomics has become one of the most enthralling fields in molecular biology as it targets the molecular link in the information chain from protein to its coding sequence and its manifestation in the form of phenotype. In contrast to the relative ease of mRNA isolation, c-DNA synthesis and analysis, protein extraction presents numerous challenges due to its heterogeneous nature, structural complexity and instability. Such features dramatically complicate their extraction, solubilization, handling, separation, and ultimately identification. Moreover no technology currently exists that is equivalent to PCR, which can amplify low abundance proteins [1].

The most critical step in any proteomic study is protein extraction and sample preparation. However, the difficulties involving plant protein extractions especially from roots are quite complicated as compared to other organisms. Root tissues are highly vacuolated with relatively low protein content. They are often rich in proteases, storage polysaccharides, lipids, phenolics and a broad array of secondary metabolites [2–4]. Such contaminants cause major obstacles for two-dimensional electrophoresis (2DE) resulting in horizontal and vertical streaking, smearing, and reduction in the number of distinctly resolved protein spots [5].

The present investigation deals with protein extraction from chickpea roots. Chickpea is the most important legume crop in India and ranks third in the world's list of important legumes. Its production is greatly hampered by different abiotic and biotic factors. Major yield loss is caused by root invading pathogens like *Sclerotium rolfsii *(collar rot), *Fusarium solani* (black root rot),* Thielaviopsis basicola* (black streak root rot), *Phytophthora *sp. (*Phytophthora* root rot), *Fusarium *sp. (*Fusarium* root rot), *Fusarium oxysporum *f.sp. ciceris (*Fusarium* wilt), and so forth. Hence, root proteins serve to be excellent target to study early signaling in plant-pathogen interaction involving root invading pathogens in particular.

Most common and basic protocols used for protein extraction from plant tissue are TCA-acetone and phenol-based extraction methods. TCA-acetone precipitation was initially developed by Damerval et al. [6]. This method increases the protein concentration and helps removing contaminants, although some polymeric contaminants are often coextracted. This appears as a problem with tissues that are rich in compounds such as soluble cell wall polysaccharides and polyphenols. Another method involves protein solubilization in phenol, with or without using SDS followed by precipitation with methanol and ammonium acetate and subsequent resolubilization in IEF (isoelectric focusing) sample buffer [5, 7, 8]. This method can efficiently generate protein extracts from resistant tissues such as wood [9], olive leaves [10], maize roots [11], and hemp roots [12], and so forth. Similar studies also suggested that phenol-based method reduces protein degradation during extraction and helps in solubilizing membrane proteins and glycoproteins [5, 13]. However, requirement of extensive time appears to be the major limitation of this method. Thus, these extraction protocols demand optimization for particular organisms, tissue or cell compartment. 

In current study attempts were made to optimize the phenol SDS method along with sonication for protein extraction from small amount of recalcitrant chickpea roots. Evaluations of other different extraction methods were also done in comparison to the optimized phenol SDS sonication method and its compatibility with high throughput method like mass spectrometry analysed. 

## 2. Materials and Methods

### 2.1. Plant Material

Experiments were performed using chickpea seeds (JG62) obtained from International Crops Research Institute for the Semi Arid Tropics (ICRISAT), Patancheru, Andhra Pradesh, India. Seeds sown in a mixture of sand and synthetic soil (1 : 1) were allowed to grow in natural green house conditions suited for the crop [14]. Roots of 15–20 days old seedlings were thoroughly washed, frozen in liquid nitrogen, and stored at −80°C prior to extraction of protein.

### 2.2. Extraction Protocols

**(A) TCA-Acetone Precipitation Method**

**Figure 1:**
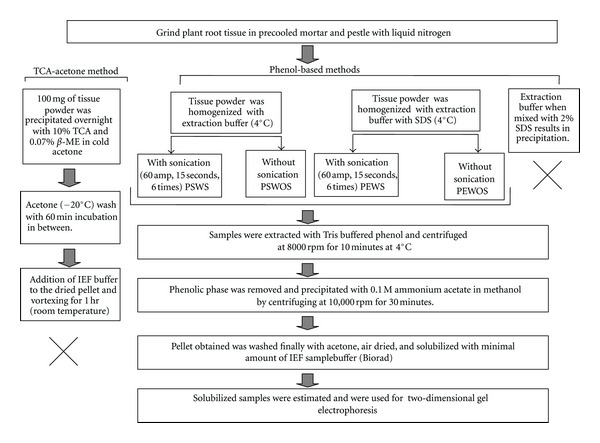
Schematic representation of extraction of protein from chickpea roots using TCA-acetone and phenol based extraction protocols.

TCA-acetone precipitation was carried out according to Damerval et al. with some modifications [6]. One gram of root material was ground in a precooled mortar in the presence of liquid nitrogen. Approximately 100–150 mg of ground tissue powder was precipitated overnight with freshly prepared 2 mL of 10% TCA, 0.07% *β*-mercaptoethanol in cold acetone. Following precipitation the set was centrifuged at 10,000 g for 15–20 min at 4°C and the supernatant discarded. The obtained pellet was rinsed twice in ice-cold acetone with 0.07% *β*-mercaptoethanol. An additional modification was introduced between the rinsing steps by incubating the sample for 60 min at −20°C [15]. The pellet was air dried, resuspended in 100 *μ*L sample buffer (8 M Urea, 2% CHAPS, 50 mM DTT, 0.2% Biolyte 3/10 Ampholyte, 0.001% Bromophenol Blue) (Biorad), and vortexed for 1 hour at room temperature. The supernatant was used for downstream analyses (Figure 1).

**(B) Phenol Extraction Method**

Phenol extraction method was used both singly and in combinations of extraction buffer and SDS along with variations of with and without sonication (Figure 1).

**(B.1) Phenol-SDS Buffer Extraction with Sonication (PSWS)**

Phenol extraction of proteins was carried out as described by Hurkman and Tanaka [7] in the presence of SDS buffer designated as phenol-SDS extraction by Wang et al. [10]. One gram of root tissue was ground in a mortar in the presence of liquid nitrogen and extracted with 3 mL of SDS buffer (30% sucrose, 2% SDS, 0.1 M Tris-Cl, 5% *β*-mercaptoethanol, and 1 mM phenyl methyl sulfonyl fluoride (PMSF), pH 8.0). The extract was sonicated 6 times for 15 seconds at 60 amps. Following sonication 3 mL of Tris buffered phenol was added to the mixture and vortexed for 10 mins at 4°C. The set was centrifuged at 8,000 g for 10 min at 4°C, phenolic phase collected and reextracted with 3 mL SDS buffer and shaken for 3–10 min. Centrifugation was further repeated using the same settings, phenolic phase collected and precipitated overnight with four volumes of 0.1 M ammonium acetate in methanol at −20°C. Precipitate obtained by centrifugation at 10,000 g for 30 min at 4°C was washed thrice with cold 0.1 M ammonium acetate and finally with cold 80% acetone. The pellet was dried and resuspended in 100 *μ*L sample buffer (Biorad) and used for further analyses.

**(B.2) Phenol-SDS Buffer Extraction without Sonication (PSWOS)**

This method was same as mentioned in case of PSWS only with the elimination of the sonication step.

**(B.3) Phenol-Extraction Buffer with Sonication (PEWS)**

One gram of frozen root tissue was homogenized in liquid nitrogen and was extracted with ice-cold extraction buffer (500 mM Tris-Cl, 50 mM EDTA, 700 mM sucrose, 100 mM KCl, pH 8.0) at 4°C. The extract was sonicated 6 times at 60 amps for 15 sec and further extracted with Tris buffered phenol as described in PSWS.

**(B.4) Phenol-Extraction Buffer without Sonication (PEWOS)**

Protein extraction was carried out in the same way as described in case of PEWS with elimination of the sonication step.

**(B.5) Phenol-Extraction Buffer with SDS**

This protocol was similar to phenol extraction method. The buffer composition was the same as mentioned in PEWS pH 8.0 with 2% SDS as an additional component. However appearance of a white precipitate following SDS addition to the basal phenol extraction buffer prevented further processing of the sample using this buffer (Figure 1).

### 2.3. Protein Quantification

Protein concentrations were quantified using the Bradford protein assay method using BSA as a standard [16].

### 2.4. Two-Dimensional Electrophoresis (2DE)

IPG strips (11 cm, 3–10 nonlinear, Readystrip, Biorad) were passively rehydrated overnight with rehydration sample buffer containing 250 *μ*g of isolated protein. IEF was carried out on PROTEAN IEF Cell (Biorad) at field strength of 600 V/cm and 50 mA/IPG strip. The strips were focused at 250 V for 20 mins, 8000 V for 2 hours 30 mins with linear voltage amplification, and finally to 20,000 volt hour with rapid amplification. Following IEF, the strips were reduced with 135 mM DTT in 4 mL of equilibration buffer (20% (v/v) glycerol, 0.375 M Tris-Cl, 6 M urea, 2% (w/v) SDS, pH 8.8) for 15 mins and alkylated with 135 mM iodoacetamide in 4 mL equilibration buffer for 15 mins. The 2DE was performed using 12% polyacrylamide gels (13.8 cm × 13.0 cm × 1 mm) in an AE-6200 Slab Electrophoresis Chamber (Atto Biosciences and Technology, China) at constant volt (200 V) for 3 hours 30 mins in Tris glycine-SDS running buffer. All 2DE gel separation was performed in triplicates for all the methods. The gels were stained with 0.1% (w/v) coomassie brilliant blue R-250 (Sigma) overnight, destained, and stored in 5% acetic acid at 4°C for further analysis.

### 2.5. Image Analysis of 2D PAGE Gels

Coomassie stained 2-D gels were visualized using Versa Doc (Model 4000) Imaging System (Biorad) and analyzed with PD Quest Advanced 2-D Analysis software (version 8.0.1, Biorad). Spots were detected automatically by the Spot Detection Parameter Wizard using the Gaussian model with standard parameters. Comparison between spot quantities across gels was performed accurately, and normalization was done using local regression model. Only spots present in each of the three replicate gels, with high and low intensity, were randomly chosen for subsequent analyses. Selected protein spots were subjected to in-gel digestion for identification by MALDI-TOF MS and MS/MS analyses.

### 2.6. MALDI-TOF MS and MS/MS Analysis and Database Search

Spots were excised from protein gels, and in-gel digestion was performed as described by Shevchenko et al. with minor modifications [17]. Proteins were digested in gel using porcine trypsin (Promega) and were extracted using 25% acetonitrile and 1% trifluoroacetic acid. One microlitre of sample and matrix (*α*-cyano-4-hydroxy cinnamic acid, HCCA) (Bruker, Daltonics) was loaded in a Anchor Chip MALDI Plate (Bruker, Daltonics). 

Mass spectra were obtained on an Autoflex II MALDI TOF/TOF (Bruker, Daltonics, Germany) mass spectrometer equipped with a pulsed nitrogen laser (*λ*-337 nm, 50 Hz). Then the spectra were analysed with Flex Analysis Software (version 2.4, Bruker, Daltonics). The processed spectra were then searched using MS Biotools (version 3.0) program, against the taxonomy of Viridiplantae (green plants) in the MSDB database using MASCOT search engine (version 2.2). The peptide mass fingerprinting parameters included peptide mass tolerance (≤100 ppm); proteolytic enzyme (trypsin); global modification (carbamidomethyl, Cys); variable modification (oxidation, Met); peptide charge state (1+) and maximum missed cleavage 1. The significance threshold was set to a minimum of 95% (*P* ≤ 0.05). The criteria used to accept protein identification were based on molecular weight search (MOWSE) score, the percentage of the sequence coverage, and match with minimum five peptides. MS/MS was performed to confirm the identification with matched peptides, selected on the basis of suitability for fragmentation (signal strength and relative isolation).

## 3. Results

### 3.1. Protein Quantification

#### 3.1.1. TCA-Acetone Precipitation Method

Protein yield using the classical TCA-acetone precipitation method was extremely low (data not shown). However a modification of incubating the sample at −20°C for 60 minutes in-between the rinsing step yielded a measurable amount of protein. Approximately seventy-three micrograms of protein were obtained from one gram of root tissue using this method (Table 1). However, when the obtained protein was subjected to electrophoresis in SDS PAGE (polyacrylamide gel electrophoresis) gel, no banding profile was visualized (data not shown). Hence, this protocol was eliminated from further downstream analysis. 

#### 3.1.2. Phenol-Based Methods

In case of phenol-based methods, protein yields obtained from PSWS, PSWOS, PEWS, and PEWOS were 600 *μ*g, 406 *μ*g, 408 *μ*g, and 300 *μ*g, respectively, (Table 1). One gram of fresh chickpea roots yielded maximum amount of protein with PSWS method as compared to protein obtained by methods PSWOS, PEWS, and PEWS.

### 3.2. Data Analysis of 2DE Gels

**Figure 2:**
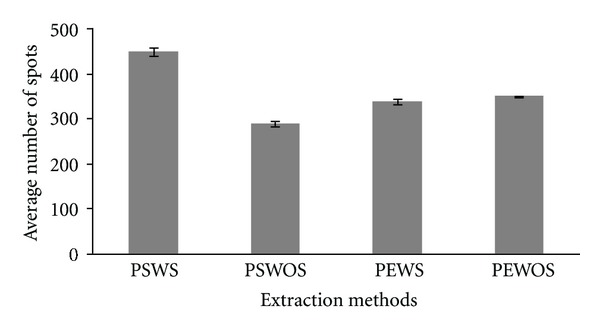
A comparative graphical representation showing the average number of protein spots detected in 2DE gels using PSWS, PSWOS, PEWS, and PEWOS protein extraction protocols.

**Figure 3:**
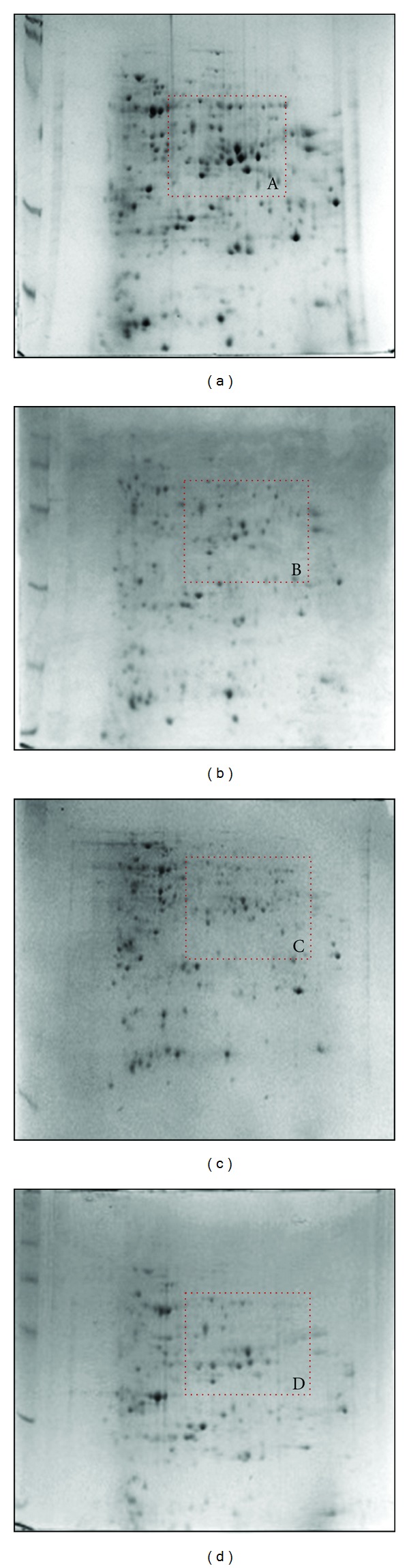
2DE profiles of chickpea root proteins of JG 62. Profile of proteins isolated using PSWS (a), PSWOS (b), PEWS (c), and PEWOS (d) extraction protocols. Inset A, B, C, D represents a close-up view of an area showing spot resolution: in PSWS (a), PSWOS (b), PEWS (c), and PEWOS (d), respectively.

**Figure 4:**
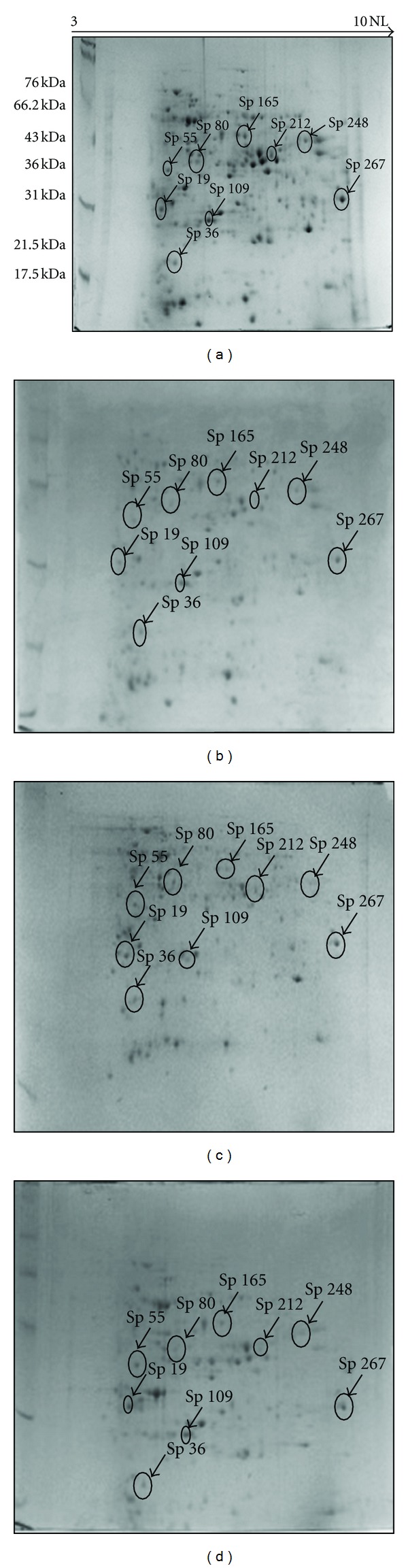
2DE profiles with marked spots selected for MALDI-TOF MS and MS/MS. (a) 2DE profile using PSWS, (b) 2DE profile using PSWOS, (c) 2DE profile using PEWS, and (d) 2DE profile using PEWOS.

**Figure 5:**
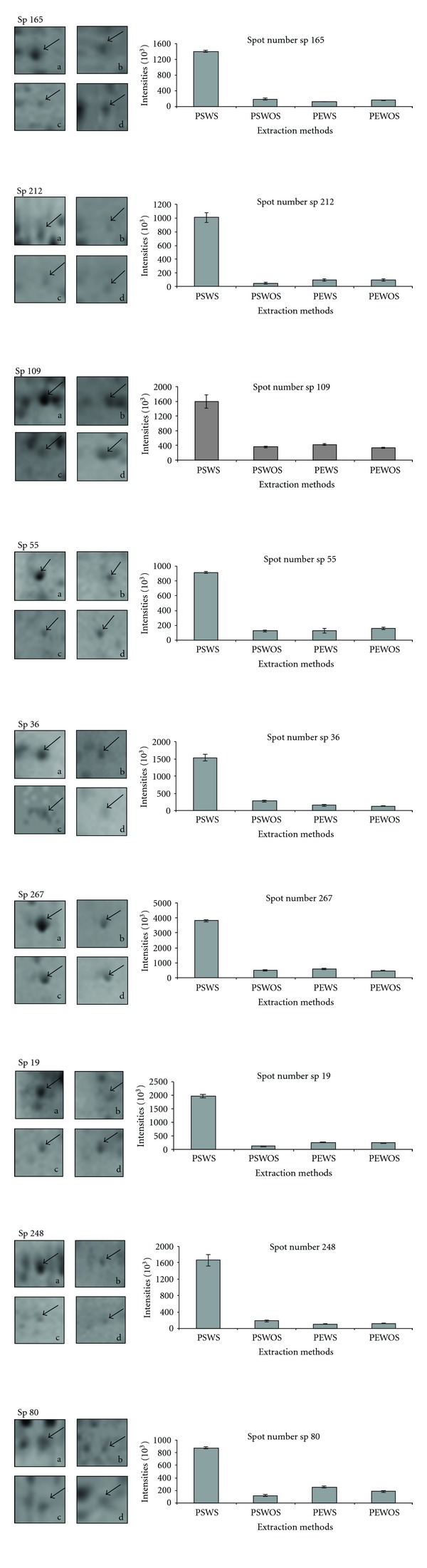
2DE gel profiles showing individual spots and their relative intensities in graphical form using PSWS, PSWOS, PEWS, and PEWOS protein extraction protocols. (a), (b), (c), (d) represent the spot obtained by PSWS, PSWOS, PEWS, and PEWOS, respectively.

The 2DE patterns of extracted protein when compared with equal amount of initial protein load revealed that protein extracted by PSWS method displayed a comparatively good resolution with lesser contamination, whereas proteins extracted with methods PSWOS, PEWS and PEWOS resolved fewer protein spots (Figure 2). Approximately 446 detectable spots (as estimated by PD Quest software) were obtained by PSWS method while 287 spots by PSWOS method, 338 by PEWS, and 348 by PEWOS method were detected (Table 2). The number of spots described in Table 2 is the average number of spots across the triplicates. In addition we also found that many spots were diffused or absent in these methods (PSWOS, PEWS, PEWOS) as indicated in the marked areas (Figures 3A, 3B, 3C, and 3D). Intensities of all the spots randomly selected for downstream MS and MS/MS were more in PSWS method as compared to other methods (Figures 4 and 5). 

### 3.3. MALDI-TOF MS and MS/MS Analysis for Protein Identification

**Figure 6:**
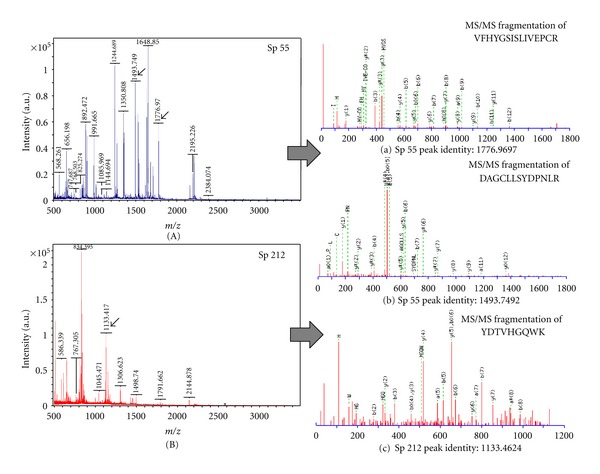
Spectral profiles obtained by MALDI-TOF MS and MS/MS. (A) MALDI spectra of sp 55 and (a), (b), show MS/MS spectra of two selected peaks of sp 55 (1776.9697 and 1493.7492). (B) MALDI spectra of sp 212 and (c) shows MS/MS spectra of the selected peak of sp 212 (1133.4624).

All the 9 spots selected for MALDI analysis (Figures 4 and 5), consisting of both less abundant (sp 36, 80, 212) and more abundant (sp 19, 55, 109, 165, 248, 267) proteins, were successfully identified and listed in Table 3 (Figure 6). Data listed in the table include assigned spot number, spot identity, protein identity (MSDB database), number of peptide matches, sequence coverage (%), MOWSE score, accession number, experimental and theoretical molecular weight and pI.

## 4. Discussion

Secondary metabolites are known to play important role in structural composition and defense of plants. These metabolites accumulate in various soluble forms in vacuoles and cause severe interference in protein extraction as well as separation in 2DE gels [18, 19]. Chickpea roots are rich in phenolic compounds like tannic acid, gallic acid, o-coumaric acid, chlorogenic acid, cinnamic acid; flavanoids, isoflavanoids like daidzein, genistein, as well as tannins, lignins, and carbohydrates [20, 21]. These compounds form hydrogen bonds with proteins. Besides they also form irreversible complexes with proteins by oxidation and covalent condensation which leads to charge heterogeneity resulting in streaking of gels [22]. Carbohydrates block gel pores causing precipitation and prolonged focusing time, which also results in loss of protein spots and streaks in the gels [15]. Although the amount of these secondary metabolites is comparatively low in etiolated tissues like roots, but low protein content and limiting tissue amounts demand for a competent protein extraction method. In our study TCA-acetone method and phenol-based method using two different extraction buffers (SDS buffer and extraction buffer without SDS) with and without sonication were evaluated. Comparison was done on the basis of protein yield, spot focusing, resolution, number of resolved spots, and also intensities of the spot and their downstream analysis using high throughput technology (MALDI/MS) of the optimized method.

Quantitative comparison of protein extracts revealed that phenol-based methods gave higher protein yield as compared to TCA-acetone method. The major reason for low protein yield in TCA-acetone method which constrained it for further downstream processing could probably be attributed to the insolubility of protein pellet in IEF buffer as compared to phenol-based methods [23]. Moreover TCA-acetone protocol is known to be effective with tissues from young plants and was found not to be the best choice for more complex tissues [5, 10, 15]. 

In case of phenol extraction, the proteins were first homogenized in two different extraction buffers; both the buffers contained sucrose which was added to create phase inversion. These buffers formed the aqueous lower phase containing carbohydrates, nucleic acid, insoluble cell debris, while the upper phenol phase contained cytosolic and membrane proteins, lipids, and pigment [15]. SDS buffer contained about 30% sucrose which helped in better phase separation as compared to extraction buffer (24%). The high pH buffers inhibit common activity of the proteases [24] and cause ionization of phenolic compounds, thus preventing them from forming hydrogen bonding with the protein [22]. It also neutralizes the acids that are released by disrupted vacuoles. PMSF and *β*-mercaptoethanol which were used in both buffers in the present study were reported to irreversibly inhibit serine protease action and act as a reducing agent which prevents protein oxidation, respectively. KCl and EDTA were used in case of extraction buffer without SDS (PEWS and PEWOS). KCl facilitates the extraction of proteins by its salting in effect and EDTA inhibits metalloprotease and polyphenoloxidase by chelating metal ions [15]. Although the salting in effect or chelation of metal ions could not improve the protein yield as compared to SDS buffer with sonication, SDS is known to act as an excellent solubilizing agent, which allows the recovery of membrane-bound proteins [10]. The solubilization of protein was found to increase with sonication as evident from the increase in protein yield and spot resolution after sonication in PSWS compared to PSWOS. Sonication results in better disruption of cell membrane and release of intracellular proteins and thus provides explanation for SDS to have efficiently solubilized the protein in PSWS method. In contrary, in case of extraction buffer, sonication could not improve protein yield or resolution, presumably due to the interference with constituents of buffer (KCl or EDTA) or due to lack of better solubilizing agent like SDS and/or both.

 The phenol used in this method was buffered to pH 8.0 to ensure that nucleic acids are partitioned to the buffer phase and not to phenol-rich phase [25], and thus proteins in phenol phase were purified and concentrated simultaneously by subsequent methanol ammonium acetate precipitation. Phenol acts as one of the strongest dissociaters known to decrease molecular interaction between proteins and other materials [15]. It can minimize protein degradation resulting from endogenous proteolytic activity [26]. Phenol extraction method though with high clean-up capacity has a little tendency to dissolve polysaccharides and nucleic acids.

We found that in PSWS method the spots obtained were well resolved and showed high intensity (Figures 3 and 5) as compared to PSWOS, PEWS, and PEWOS. About 25% unique spots were obtained in PSWS and the rest 75% spots though existed in PSWOS, PEWS, and PEWOS, however, resolved with variable clarity. Streaking was absent in all the gels. We could see that the difference in number of spots between PSWS and PSWOS was more as compared to PEWS and PEWOS, which confirmed that the effectivity of SDS increased in presence of sonication. However in the latter case (PEWS, PEWOS) sonication did not have much influence.

Improvisation of the extraction buffer was also made by adding 2% SDS, which resulted in precipitation. Interference between constituents of the extraction buffer and SDS was assumed to be the cause of such precipitation. However further experimentation needs to be performed for confirmation of such predictions. 

All protein spots selected for MALDI-TOF/MS and MS/MS from PSWS resulted in successful identification. High intense spot like sp 55, (fructokinase-like protein) and less intense spot like sp 212, (glyceraldehyde 3-phosphate dehydrogenase) both resulted in high quality spectra with low background noise (Figure 6). These results further indicated the compatibility of PSWS method with both MS and MS/MS and its reliability for downstream processing.

## 5. Conclusion

The present study emphasizes PSWS as the optimized phenol-based method for chickpea root protein extraction. This method successfully isolated high quality protein suitable for downstream processing. Hence, the data obtained projects this protocol as an effective and efficient one that could be applied for other recalcitrant leguminous root tissues as well. Nevertheless, it should be kept in mind that one generalized protein extraction protocol applicable for global protein profiling of variable tissues irrespective of their origins though theoretically conceivable, but fails to meet practical feasibility. 

## Figures and Tables

**Table 1:** Protein yield/fresh weight of root tissue (*μ*g/gm) using Bradford method.

Methods	Protein yield (*μ*g/gm)
PSWS	603 ± 6.08
PSWOS	406 ± 5.77
PEWS	302 ± 5.51
PEWOS	408 ± 7.64
TCA	73 ± 2

**Table 2:** Total number of spots using different methods.

Methods	Average number of spots
PSWS	446 ± 9.07
PSWOS	287 ± 6.43
PEWS	338 ± 6.11
PEWOS	348 ± 1.53

**Table 3:** Proteins identified by MALDI-TOF MS analyses.

S no.	Spot ID.	Protein identity	Peptides matched	Sequence coverage (%)	MOWSE score	Accession number (NCBI)	Mr(kDa)/pI experimental (theoretical)	Plant species
1	sp 165	NADP specific isocitrate dehydrogenase	10	17%	70	Q9XGU7_ORYSA	46.4/6.29 (46.0/6.0)	*Oryza sativa *
2	sp 212	Glyceraldehyde 3 phosphate dehydrogenase	9	24%	86	Q6K5G8_ORYSA	36.716/7.68 (37/6.5)	*Oryza sativa *
3	sp 109	Triose phosphate isomerase	6	20%	71	Q38IW8_SOYBN	27.4/5.87 (25/5.5)	*Glycine max *
4	sp 55	Fructokinase-like protein	9	40%	94	Q8LPE5_CICAR	26.26/5.03 (35.5, 4.5)	*Cicer arietinum *
5	sp 36	ATP synthase (subunit D chain)	13	36%	88	ATPQ_ARATH	19.4/5.09 (20/5.0)	*Arabidopsis thaliana *
6	sp 267	Porin of Pea, channel protein	2	11%	134	T12558	29.7/8.56 (30/9.5)	*Phaseolus coccineus *
7	sp 19	Plasma membrane intrinsic polypeptide	10	38%	74	Q9SMK5_CICAR	23.3 /4.95 (24.5/5.0)	*Cicer arietinum *
8	sp 248	Unidentified protein	11	35%	80	CAA06491	22.12/9.91 (44.0,9.0)	*Cicer arietinum *
9	sp 80	Putative pyruvate dehydrogenase E1 beta subunit isoform 1 protein	2	6%	55	Q6Z1G7_ORYSA	40.2/5.25 (38.5/5.3)	*Oryza sativa *
